# The Effects of Family Functioning and Psychological Suzhi Between School Climate and Problem Behaviors

**DOI:** 10.3389/fpsyg.2020.00212

**Published:** 2020-03-10

**Authors:** Ting Zhang, Zhi Wang

**Affiliations:** Faculty of Psychology, Research Center of Mental Health Education, Southwest University, Chongqing, China

**Keywords:** perceived school climate, psychological suzhi, family functioning, problem behaviors, adolescence

## Abstract

Problem behaviors have always been a hot topic in the field of adolescent research. It is particularly important to study how problem behaviors are developed. Empirical evidence examining problem behaviors has shared the premise that perceived school climate and family functioning play a role in the development of problem behaviors in adolescents. However, it is less clear whether the interaction of perceived school climate and family functioning can predict problem behaviors in adolescents and which mechanisms within the process it might affect. The present study developed a moderated mediation model to investigate the relationship between perceived school climate, family functioning, psychological suzhi, and problem behaviors in early adolescents. Participants were 1,072 Chinese junior high school students who completed the Perceived School Climate Questionnaire, the Strengths and Difficulties Questionnaire, the Psychological Suzhi Questionnaire for Middle School Students, and the Family APGAR scales. Data were analyzed using IBM SPSS Statistics version 22.0, including descriptive statistics and correlation analysis. The mediating effect and moderating effect were tested by SPSS PROCESS. Results showed that there was a significant negative correlation between perceived school climate and problem behaviors and a partial mediating role of psychological suzhi between perceived school climate and problem behaviors. Moreover, the influence of perceived school climate on psychological suzhi was moderated by family functioning. Indirect effects were significant in participants with high versus low family functioning. There was an interaction between family and school, and psychological suzhi played an important role between environment and adolescent behaviors. This study validates the combined effect of family systems, school systems, and personal systems on problem behaviors and has certain guiding significance for the prevention and intervention of problem behaviors among adolescents.

## Introduction

Problem behavior, which refers to adolescents’ learning and social maladjustment in changing environment, has been demonstrated to affect adolescents’ physical and mental health (Barber and Xia, 2013; Ryan et al., 2013) and to increase the likelihood of problem behaviors, such as fighting, smoking, and drinking in the future (Huang et al., 2012; Yang et al., 2013). Problem behavior may even become a risk factor affecting social stability (Fischer et al., 2012; Yun et al., 2016). Empirical evidence examining school climate and adolescent adjustment has shared the premise that perceived school climate plays a role in the development of problem behaviors in adolescents (Wang and Fredricks, 2014; Bao et al., 2015). Family functioning is also an important environmental variable affecting adolescent development (Owrangi et al., 2011; Wildeman et al., 2016). However, in contrast to the impact of individual variables on adolescent development, it is less clear whether perceived school climate and family functioning interact to predict problem behaviors in Chinese adolescents and which mechanisms within this process it might affect (considering individual variables within Chinese cultural characteristics, e.g. psychological suzhi). Early adolescents confront a series of new educational and emotional demands as they enter junior high school, which places some of them at greater risk of developing problem behaviors (Reinke and Herman, 2002; Connell et al., 2009). Therefore, we tested the following hypotheses that (1) perceived school climate will play a role in problem behaviors in Chinese early adolescents, (2) psychological suzhi will mediate the relationship between perceived school climate and problem behaviors, and (3) family functioning will moderate the path from perceived school climate to psychological suzhi.

### Perceived School Climate and Problem Behaviors

Grounded in ecological theories of development, the ecological model posits that the development of adolescent problem behaviors is a complex and dynamic process involving interactions between adolescent characteristics and features of the environments in which they are situated (Crosnoe et al., 2002; Wang, 2009). School experience is undoubtedly an important microenvironment that has a profound impact on adolescent development, as they spend more than two-thirds of their waking time at school (Eccles and Roeser, 2011). Over the past 20 years, a growing body of research has shown the impact of student perceptions of school climate on their psychological and emotional adjustment (Reaves et al., 2018), as well as their behavioral adjustment (LaRusso et al., 2008; Wang and Dishion, 2012). Furthermore, empirical studies with this focus have repeatedly confirmed that adolescents’ positive perceptions of school climate have significant concurrent and prospective associations with fewer behavioral problems (Way et al., 2007; Bao et al., 2015). Therefore, it is hypothesized that perceived school climate will negatively predict the problem behaviors of Chinese early adolescents (H1).

### Mediating Role of Psychological Suzhi

Psychological suzhi, a native Chinese concept, is an essential, implicit mental quality that has differences and connections with resilience and affects adaptive and developmental behavior (Zhang et al., 2000; Zhang, 2003; Wang, 2014). Psychological suzhi could be regarded as three dimensions (cognitive quality, individuality, and adaptability) and has been proven to mutually verify and support the Dual-Factor Model of Mental Health (Keyes, 2014; Wang et al., 2016). Psychological suzhi plays a central role in the individual’s quality structure and is the internal basis and motivation of individual psychological and behavioral development. As an endogenous psychological variable, suzhi will inevitably affect individual behavior because behavior and psychology are inseparable. Furthermore, considerable evidence indicates that psychological suzhi influences student adjustment across multiple domains (Liu et al., 2016; Zhang D. J. et al., 2017). Previous studies have demonstrated that psychological suzhi is significantly inversely correlated with problem behavior in children and adolescents (Wu et al., 2015; Liu et al., 2017b) and that higher levels of psychological suzhi significantly reduce the likelihood of internalizing problem behaviors, including depression and anxiety in children and adolescents (Hu and Zhang, 2015; Su and Zhang, 2015; Liu et al., 2017a). These studies suggest that higher levels of psychological suzhi predict lower levels of problem behaviors in early adolescents.

In contrast, according to the formation mode of psychological suzhi, the individual’s selective internalization of external stimulation is under certain social conditions through interaction with experienced adults (e.g. parents and teachers) and peers (Zhang et al., 2013). Psychological suzhi is both a stable mental quality and a plastic entity that can be shaped by the external environment especially for children and adolescents (Zhang et al., 2011). Moreover, according to the ecological systems theory and sociopsychological models of problem behavior, perceived school climate, an important environmental variable, can influence a student’s behavioral outcome through individual variables (such as psychological suzhi; Yu and Ge, 2010; Peter et al., 2017). Empirical evidence indicates that school climate can affect the development of psychological suzhi (Nie, 2016). There is also research showing that psychological suzhi plays a mediator role between parental emotional warmth and problem behavior in primary school students (Wu et al., 2015; Pan et al., 2017). Therefore, we investigate whether psychological suzhi plays an intermediary role between perceived school climate and problem behaviors in early adolescents (H2).

### Moderating Role of Family Functioning

Family functioning is one of the important factors in evaluating the quality of the family environment, including role allocation, communication, emotional response, problem solving ability, and behavior control among family members (Zaider et al., 2020). The family function aims to integrate various characteristics of the family, consider the family as a system, and examine the overall function of the family system. It is an important indicator of an individual’s family system operation and is closely related to mental health in adolescents (Shek, 2002). The theory of psychological suzhi assumes that suzhi forms through interactions between individuals and proximal environments (Zhang et al., 2011), and family will have an important impact on the development of students’ psychological suzhi because family is the most direct and recent microenvironment affecting children’s psychological development (Zeleke, 2013). Some researchers have pointed out that family functioning has been the key to understanding adolescent psychological problems (Milburn et al., 2017). Moreover, research has shown that family functioning plays an important role in the development of psychological suzhi (Liang et al., 2018).

In recent years, research on the development of psychopathology has begun to focus on the interactive effects of different developmental backgrounds, such as the effects of school and family on adolescent development (Crosnoe, 2004; DeLay et al., 2013). It is more beneficial to individual development to intervene on various factors, rather than to intervene on only a single factor from a practical perspective (Luthans et al., 2006; Pervanidou et al., 2019). Developmental system theory states that school and family not only act independently on youth development, but they also interact and connect (Lakind et al., 2015). Increasingly, studies have also confirmed interactions between different development backgrounds (Luthans et al., 2015; Zhang Y. et al., 2017). Furthermore, family functioning has been determined to be an important environmental factor for moderating the relationship between school and mental health (DeLay et al., 2013; Yang et al., 2016). Considering that psychological suzhi and mental health are related by “essence” and “surface” (Zhang and Wang, 2012), the present study hypothesizes that family functioning will play a moderator role between perceived school climate and psychological suzhi (H3).

### Current Study

**FIGURE 1:**
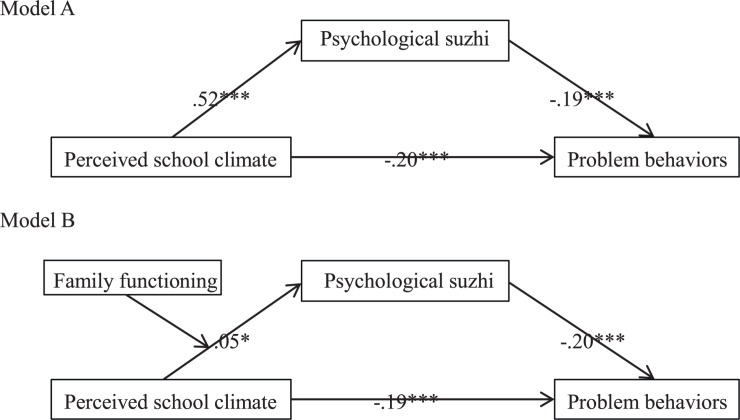
Mediation model **(model A)** and moderated mediation model **(model B)**. *^∗^p* < 0.05, ^∗∗∗^*p* < 0.001.

The present study aims to figure out (1) the effect of perceived school climate on problem behaviors in Chinese early adolescents; (2) the intermediary mechanism of psychological suzhi; and (3) the moderating role of family functioning between perceived school climate and psychological suzhi by building a moderated mediating model. We focus on the interaction between perceived school climate and family functioning because the existing literature on problem behaviors overlooks interactions with environment variables. Additionally, a Chinese native concept, psychological suzhi, has been introduced to this study to better examine the adaptability of the relationship between school climate and problem behavior in Chinese adolescents. Moreover, “Xiaoshengchu” (the transition from elementary school to junior high school) is a particularly challenging period for junior high school students, and the school environment (e.g. teacher support, peer support, and autonomy opportunities) is critical to the successful transition of early adolescents (Martínez et al., 2011). Figure 1 depicts the proposed model.

## Materials and Methods

### Participants

Participants were 1,072 young adolescent students (aged 13.03 ± 0.997 years) recruited from four junior high schools in Chongqing, China. Boys and girls comprised 493 and 553 participants, respectively, with 26 participants reporting no gender. All participants were of Han ethnicity.

### Measures

#### Problem Behaviors

The Strengths and Difficulties Questionnaire was used to screen for adolescent problem behaviors. The scale, which developed in 1997 by Goodman, was revised, made more extensive, and formulated with Shanghai norms by the Shanghai Mental Health Center. The questionnaire includes 20 items divided evenly across four dimensions: emotional symptoms, conduct problems, hyperactivity, and peer interaction. Four items use reverse scoring. Each entry is scored on a scale of 0 to 2, where 0 points = non-conformity, 1 point = somewhat consistent, and 2 points = full compliance. The Cronbach’s α coefficient for the entire questionnaire was 0.634. Structural validity [comparative fit index (CFI) = 0.726, Tucker–Lewis Index (TLI) = 0.680, χ^2^/df = 7.44, root mean squared error of approximation (RMSEA) = 0.078, standardized root mean square residual (SRMR) = 0.088] were not in line with the standards of psychometrics.

#### Perceived School Climate

We used the 25-item Perceived School Climate Questionnaire designed by the American Center for Interventional Research and Development (Felner et al., 1997) and adapted by Jia et al. (2009) to measure school climate. The measure assesses adolescents’ perceptions of school climate on three dimensions: teacher support, student–student support, and opportunities for autonomy. All items have a four-point response scale (1 = never, 4 = always). The negatively keyed items were reverse-coded, and mean subscale scores were used, with higher values indicating better perceived school climate. Previous research has indicated that this measure has internal consistency and convergent and discriminant validity (Jia et al., 2009). The Cronbach’s α coefficient for the entire questionnaire was 0.811. Structural validity [CFI = 0.879, TLI = 0.864, χ^2^/degrees of freedom (df) = 4.16, RMSEA = 0.043, SRMR = 0.034] was not all in line with the standards of psychometrics.

#### Psychological Suzhi

We examined psychological suzhi using the Psychological Suzhi Questionnaire for Middle School Students (PSMQ; Hu et al., 2017), which was developed and validated based on a bifactor model (Reise et al., 2013; Vecchione et al., 2014). The questionnaire consists of three dimensions: cognitive quality, individuality, and adaptability, and each dimension includes eight items. Participants indicated their responses to each item on a five-point Likert scale (1 = totally disagree, 5 = totally agree). The PSMQ has good internal consistency and reliability for the total scale (α = 0.91; Hu et al., 2017). In the present study, the internal reliability was α = 0.893 for the total scale. Structural validity (CFI = 0.932, TLI = 0.919, χ^2^/df = 7.63, RMSEA = 0.046, SRMR = 0.034) was in line with the standards of psychometrics.

#### Family Functioning

The family APGAR (Smilkstein, 1978) assesses the extent to which individuals are satisfied with their family functioning. It is a five-item scale that measures five domains of family functioning: adaptation, partnership, growth, affection, and resolve. Responses indicate on a three-point scale the extent to which the statements are true of their family. Total scores range from 0 (hardly ever) to 2 (almost always), with higher scores revealing higher levels of satisfaction with family functioning. The reliability of the APGAR is satisfactory [Cronbach’s α = 0.80 with a 2-week test–retest reliability coefficient of 0.83 (Smilkstein et al., 1982)]. In this study, the Cronbach’s α coefficient was 0.772. Structural validity (CFI = 0.983, TLI = 0.967, χ^2^/df = 4.18, RMSEA = 0.054, SRMR = 0.021) was in line with the standards of psychometrics.

## Results

### Descriptive Statistics

Table 1 shows the means, standard deviations, and correlations among perceived school climate, problem behaviors, psychological suzhi, and family functioning. A correlation analysis measured the degree of correlation among the variables, and we found that the magnitude and direction of the correlation coefficients were in line with our expectations.

**TABLE 1:** Descriptive statistics and correlations among study variables.

	Age	Gender	PSC	PS	FF	PB
Age	–					
Gender	−0.06*	–				
PSC	−0.22**	0.12**	–			
PS	−0.19***	0.09**	0.51**	–		
FF	−0.07*	0.03	0.41**	0.32**	–	
PB	0.09	–0.024	−0.29**	−0.30*	−0.15***	–
M	13.03	0.53	2.97	3.52	1.41	0.64
SD	1.00	0.50	0.39	0.55	0.49	0.25

N = 1072. * p < 0.05, ** p < 0.01, *** p < 0.001. PSC, perceived school climate; PS, psychological suzhi; FF, family functioning; PB, problem behaviors; SD, standard deviation.

### Mediating Effect of Psychological Suzhi

We conducted mediation analysis (Preacher and Hayes, 2004) to test whether psychological suzhi mediated the relationship between perceived school climate and problem behaviors (see Model A in Figure 1). All variables were standardized. Gender and age were entered as covariates. Bootstrap estimates were based on 5,000 bootstrap samples. Table 2 reports statistical mediation results. The results indicate that psychological suzhi partly mediated the relationship between perceived school climate and problem behaviors. Approximately 33.33% of the total variance was accounted for by the indirect effect.

**TABLE 2:** Summary of mediation results.

		Model summary						
*Y*	*X*	*R*	*R*^2^	*F*	*B*		SE	95% CI
On PS	PSC	0.51	0.27	125.04	0.52***		0.03	0.46, 0.58
	Gender				0.03		0.03	−0.03, 0.09
	Age				−0.14**		0.05	−0.23, −0.05
On PB	PS	0.35	0.12	35.45	−0.19***		0.03	−0.26, −0.13
	PSC				−0.20***		0.03	−0.27, −0.13
	Gender				0.02		0.03	−0.04, 0.08
	Age				0.01		0.05	−0.08, 0.11

**Effect**		***B***		**Boot SE**		** Boot LLCI**		**Boot ULCI**

Direct		−0.20		0.03		−0.27		−0.13
Indirect		−0.10		0.02		−0.14		−0.07

N = 1072. ** p < 0.01, *** p < 0.001. PSC, perceived school climate; PS, psychological suzhi; FF, family functioning; PB, problem behaviors.

### Moderating Effect of Family Functioning

We examined whether the strength of the mediated relationships was contingent on family functioning. Specifically, we tested whether family functioning moderated the effect of perceived school climate on psychological suzhi in the mediation models (see Model B in Figure 1). Our moderated mediation analysis followed Preacher and Hayes (2004) guidelines. All variables were standardized, and gender and age were entered as covariates. The analysis results are shown in Table 3.

**TABLE 3:** Moderated mediation analysis results with psychological suzhi as mediator.

Predictor	Mediator variable model: DV = PS					
	*B*	SE		*t*	*p*	95% CI
PSC	0.46	0.03		14.41	<0.001	0.40, 0.52
FF	0.15	0.03		4.99	<0.001	0.09, 0.21
PSC × FF	0.05	0.03		2.10	<0.05	0.00, 0.10
Gender	0.03	0.03		1.25	0.21	−0.02, 0.09
Age	−0.15	0.05		−3.30	<0.01	−0.24, −0.06

*R*^2^	0.28					
*F*	82.47***					

**Predictor**	**Dependent variable model: DV = PB**					
	***B***	**SE**		***t***	***p***	**95% CI**

PSC	−0.19	0.03		−6.00	<0.001	−0.26, −0.13
PS	−0.20	0.03		−5.70	<0.001	−0.27, −0.13
Gender	0.02	0.03		0.68	0.50	−0.04, 0.08
Age	0.01	0.05		0.31	0.76	−0.08, 0.11

*R*^2^	0.12					
*F*	35.45***					

**FF**	**Conditional indirect effect at specific values of family functioning**					
	***B***		**SE**		**95% CI**	

−1 SD (−0.95)	−0.08		0.02		−0.12, −0.05	
*M* (0.04)	−0.09		0.02		−0.13, −0.05	
+1 SD (1.04)	−0.10		0.02		−0.14, −0.06	

N = 1072. *** p < 0.001. PSC, perceived school climate; PS, psychological suzhi; FF, family functioning; PB, problem behaviors; SD, standard deviation.

**FIGURE 2:**
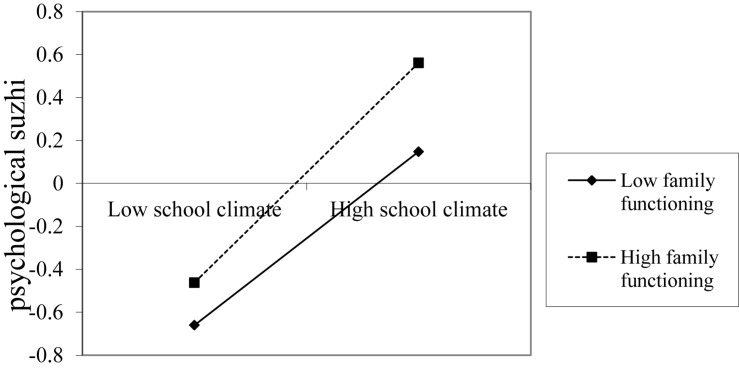
Moderating effect of family functioning on the prediction of psychological suzhi.

To understand the moderating effect, we conducted simple effect analysis (Aiken and West, 1991). For perceived school climate and psychological suzhi, the mean of family functioning ± 1 standard deviation was considered to draw a simple effect analysis (Figure 2).

## Discussion

### Perceived School Climate and Problem Behaviors

The present study found a significant negative correlation between perceived school climate and adolescent problem behaviors, meaning individuals who experienced a positive perceived school climate were less likely to exhibit problem behaviors. This finding is consistent with the existing research (Way et al., 2007; Wang and Fredricks, 2014). According to Bronfenbrenner (1986), children problem behaviors are shaped by both the characteristics of the student and the environment closest to him or her (Crosnoe et al., 2002). As individuals enter adolescence with an increasing sense of independence, school plays a more important part in adolescents’ lives than parents because they spend more time at school. School is undoubtedly an important microenvironment that has a profound impact on adolescent development (Eccles and Roeser, 2011). A positive climate that embraces warmth and support will help teenagers reduce problem behaviors (Bierman et al., 2010). Conversely, when adolescents are exposed to a negative environment, they naturally face more challenges and threats, which increase the potential for problem behaviors to arise.

### Perceived School Climate, Psychological Suzhi, and Problem Behaviors

The current study found that psychological suzhi plays a partial mediating role between perceived school climate and adolescent problem behaviors. This finding is consistent with the theoretical expectation that perceived school climate both directly predicts adolescent problem behaviors and influences the occurrence of problem behaviors through adolescents’ psychological suzhi. These results are consistent with those of other related studies (Pan et al., 2017). It is generally believed that external causes become operative through internal causes because external causes are conditions of change and internal causes are the basis for change (Nie, 2016). Therefore, perceived school climate can influence problem behaviors through psychological suzhi.

Our findings suggest that when the perception of school climate is more positive adolescents display more high-level psychological suzhi. This result may be explained by the concept and formation mode of psychological suzhi. It is a basic, stable, and derivative psychological quality that is based on physiological conditions and internalized by external stimuli. Psychological suzhi can form through interactions between individuals and school environments (Zhang et al., 2011) because school plays a more important role when adolescents enter junior high. This finding, the existence of a mediator role, also shows a significant negative correlation between psychological suzhi and adolescent problem behaviors. In other words, individuals with high psychological suzhi reported fewer problem behaviors. This result is consistent with those of previous studies (Wu et al., 2015; Liu et al., 2017b). According to the connotation of psychological suzhi as an essential, implicit mental quality that affects adaptive and developmental behavior, it contains three personal quality dimensions: cognitive quality, individuality, and adaptability (Zhang et al., 2000; Zhang, 2003). Psychological suzhi is the internal basis and motivation of individual psychological and behavioral development, and as an endogenous psychological variable, it will inevitably affect individual behavior because behavior and psychology are inseparable.

### Perceived School Climate, Family Functioning, and Psychological Suzhi

This study found that family functioning plays a moderating role between perceived school climate and psychological suzhi in Chinese early adolescents. This finding is consistent with the theoretical expectation that perceived school climate and family functioning interact and can positively predict psychological development. These results are consistent with those of other related studies (Leung et al., 2010; Hazel et al., 2014). Our finding suggests that positive school climate can promote the development of adolescents’ psychological suzhi more easily when family functioning works well. The finding also supports the interaction-enhanced model, which is “hypothesis promoting.” Families are the social and cultural environment in which individuals first live and grow and also provide the foundation for young people’s future psychological development. Previous research has shown that family functioning can promote the development of psychological suzhi (Liang et al., 2018). Furthermore, the family system is one of the most important protective factors affecting the development of adolescents (Grant et al., 2006). When family functioning works well, adolescents feel more love and concern from their parents, and their needs can be fully expressed and satisfied, leading to a high-level psychological suzhi and a tendency for adolescents to form positive perceptions and explanations regarding the outside world. The common communication between parents and adolescents also helps adolescents to better cope with the difficulties and challenges of their external environment (Anderson et al., 2019), especially when facing a negative perceived school climate. Previous studies have generally focused on the impact of schools and peers on individual development in adolescence, ignoring the role of family. However, family and school are two closely related systems, and the individual can achieve optimal development only with good interaction between the family and school (Bronfenbrenner, 1979).

### Implications and Limitations

The current study expands the understanding of the mechanisms underlying the effect of perceived school climate on problem behaviors in Chinese early adolescents by supporting the mediating role of psychological suzhi and the moderating role of family functioning. Furthermore, this study is the first to investigate the role of psychological suzhi within the domain of school and family in Chinese early adolescents. In clinical practice, the results of this study also provide parents, teachers, and students themselves with inspiration. First, we should be mindful of young people’s psychological suzhi and consciously cultivate it. Second, teachers should provide more care for students (Zhang et al., 2019) and guide positive peer relationships to foster a positive school climate for early adolescents. Third, parents should create a warm family atmosphere, promote effective parent–child communication, and cultivate intimate parent–child relationships to provide strong support for young people’s growth and development.

Despite the theoretical and practical implications discussed above, the current study has several possible limitations. First, although we used different self-report methods and formats to measure perceived school climate, psychological suzhi, family functioning, and problem behaviors, one-sided self-report responses may have affected the level of the variables and may also have biased the study’s internal validity and reduced or prevented common-method covariance. To overcome this limitation, future research should attempt to use more objective measures to test the accuracy of the moderated mediating model. Second, we used a cross-sectional design, which can only provide an objective description of the phenomenon and the impact trends and cannot test possible causal relationships. Therefore, future research should use longitudinal or rigorous experimental designs to conduct more in-depth and meticulous research on the influencing factors and mechanisms of adolescents’ problem behaviors.

## Conclusion

The current study expands our understanding of the mechanisms underlying the effect of perceived school climate on problem behaviors in Chinese early adolescents by supporting the mediating role of psychological suzhi and the moderating role of family functioning. Furthermore, this study is the first to investigate the role of psychological suzhi within the domain of school and family in Chinese early adolescents. In clinical practice, the results of this study also provide parents, teachers, and students themselves with inspiration. First, we should be mindful of young people’s psychological suzhi and consciously cultivate it. Second, teachers should provide more care for students (Zhang et al., 2019) and guide positive peer relationships to foster a positive school climate for early adolescents. Third, parents should create a warm family atmosphere, promote effective parent–child communication, and cultivate intimate parent–child relationships to provide strong support for young people’s growth and development.

## Data Availability Statement

The datasets generated for this study are available on request to the corresponding author.

## Ethics Statement

The studies involving human participants were reviewed and approved by the Ethics Committee for psychological research at the authors’ institution. Written informed consent to participate in this study was provided by the participants’ legal guardian/next of kin.

## Author Contributions

Both authors listed have made a substantial, direct and intellectual contribution to the work, and approved it for publication. ZW put forward a theoretical idea and collected the data. TZ analyzed the data and wrote it into the article.

## Conflict of Interest

The authors declare that the research was conducted in the absence of any commercial or financial relationships that could be construed as a potential conflict of interest.
